# Validity evidence for a virtual multiple mini interview at a pharmacy program

**DOI:** 10.1186/s12909-023-04521-9

**Published:** 2023-08-03

**Authors:** Sarah Hammond, Jacqueline E. McLaughlin, Wendy C. Cox

**Affiliations:** 1 School of Social Work, UNC Chapel Hill, Chapel Hill, NC USA; 2 Division of Practice Advancement and Clinical Education, Director, Center for Innovative Pharmacy Education and Research, UNC Eshelman School of Pharmacy, UNC Chapel Hill, Chapel Hill, NC USA; 3 Division of Practice Advancement and Clinical Education, Associate Dean for Admissions and Accreditation, UNC Eshelman School of Pharmacy, UNC Chapel Hill, Chapel Hill, NC USA; 4 UNC Eshelman School of Pharmacy, University of North Carolina at Chapel Hill, Chapel Hill, NC 27599 USA

**Keywords:** Multiple mini interview, Virtual, Validity, Remote, Assessment, Pharmacy

## Abstract

**Background:**

Numerous health professions schools have transitioned to virtual admissions interviews in recent years. While some research suggests that virtual multiple mini-interviews (vMMIs) are feasible, acceptable, and more affordable, there is a paucity of research concerning the validity of this approach. The purpose of this study was to examine the validity and reliability of vMMIs and explore differences in performance between vMMI and in-person MMIs.

**Methods:**

Data were collected for two years of in-person MMIs and two years of vMMIs at a pharmacy program/school in the United States. An exploratory factor analysis (principal components analysis) with varimax rotation and Kaiser rule (i.e. retaining factors with eigenvalue > 1.0) was used to explore the construct validity of the vMMI data. Pearson correlation was used to examine correlations between vMMI stations and Cronbach alpha was used to determine the internal consistency of each station. Independent *t*-tests were used to examine differences between in-person MMI and vMMI scores. Cohen’s d was used to determine effect sizes.

**Results:**

Four hundred and thirty-eight (42.69%) candidates completed an in-person MMI and 588 (57.31%) completed a vMMI. Factor analysis indicated that each vMMI station formed a single factor with loads ranging from 0.86 to 0.96. The vMMI stations accounted for most of the total variance, demonstrated weak to negligible intercorrelations, and high internal consistency. Significant differences between in-person and vMMI scores were found for the teamwork-giving, teamwork-receiving, and integrity stations. Medium effect sizes were found for teamwork-giving and teamwork-receiving and a small effect size was found for integrity.

**Conclusions:**

Initial evidence suggests that the vMMI is a valid and reliable alternative to in-person MMIs. Additional research is needed to examine sources of differences in rating patterns between the two approaches and identify strategies that align with institutional priorities for recruitment and admissions.

## Background

Health professions schools spend a considerable amount of time and resources developing processes for student selection. Numerous studies indicate that multiple mini-interviews (MMI) are a valid and reliable method for assessing prospective health professions students [1–4]. Commonly assessed attributes include interpersonal skills (e.g., empathy, communication, integrity, adaptability) as well as ethical reasoning and situational judgment [1, 5–7]. MMIs have been utilized by various professions for over a decade and continue to be a useful admissions tool for assessing candidates while reducing bias [1, 8, 9].

Although MMIs were initially designed as an in-person circuit, the COVID-19 pandemic forced many institutions to adopt virtual MMI (vMMI) designs. Initial research suggests that vMMIs are feasible, functional, satisfactory, and – in some cases - preferable. A case study by Cleland and colleagues, for example, found vMMIs to be a feasible alternative to in-person MMIs with appropriate planning and organization [10]. Candidates and interviewers have reported high levels of satisfaction with vMMI participation [11, 12]. Further, medical students and residents have agreed that health professions programs should offer a virtual interview option with medical students preferring the virtual setting over in-person opportunities [13].

Given their remote nature, vMMIs may also help increase access to health professions schools. Numerous studies have discussed its potential to increase access for geographically diverse candidates and those from lower socio-economic backgrounds [14–16]. In-person interviews can include considerable travel and opportunity costs for candidates, including lodging and missed classes. Studies suggest that vMMIs can reduce admissions-related costs for candidates and interviewers [7, 17].

Despite the benefits of vMMIs for assessing candidates for health profession programs, there is a paucity of research exploring validity evidence for this approach [11, 17, 18]. Therefore, the purpose of this study was to explore the outcomes of a vMMI, including evidence of validity and reliability, as well as comparisons with candidate performance from in-person MMIs.

## Methods

### Multiple mini-interview design

In person MMIs were implemented at the University of North Carolina (UNC) at Chapel Hill Eshelman School of Pharmacy in 2013 [1]. The School’s MMI included seven stations, each designed to evaluate a specific construct: teamwork (giving instructions); teamwork (receiving instructions); integrity; adaptability; empathy; critical thinking; and why UNC. Candidates were allotted two minutes to read the station scenario before entering the room for six minutes to discuss it with a trained interviewer. The interviewer stayed in their station evaluating the same construct for the entire MMI and used standardized probing questions during the interview as needed. Research examining the psychometric properties of the School’s MMI model found strong evidence of validity, reliability, and acceptability [1, 9].

In response to the COVID-19 pandemic, the School transitioned to vMMIs conducted via Zoom in 2020. The 2020–2021 vMMI included the same seven stations, with candidates rotating through Zoom breakout rooms. Each interviewer remained in the breakout room for the entire vMMI. Candidates were placed in the breakout room by a support staff, provided two minutes to read a scenario shared by the interviewer via “share screen” function, and given six minutes to discuss the scenario with the interviewer. Candidates were asked to sign a confidentiality statement agreeing that they would not share the scenario. The interviewer used standardized probing questions designed to elicit the construct of interest during the interview. The vMMI teamwork station required modifications since the in-person design involved two candidates facing opposite directions (i.e., back-to-back) and either providing or receiving instructions for drawing an object. This station could not be easily reproduced in the virtual environment, so it was separated into two Zoom breakout rooms; in each vMMI room, the candidate was paired with a current PharmD student who either gave or received instructions with the candidate. Interviewers noted that the two-station virtual format was logistically awkward, and they found it difficult to differentiate students. Due to lack of perceived value for the two-station virtual format, teamwork was consolidated into one station in 2021–2022 and the candidate was asked to collaborate with a current PharmD student to rank order items in response to a scenario.

Candidates were evaluated by the interviewers with the same rubric used for in-person MMIs, which was designed specifically for the station construct of interest. Each candidate was rated on a 10-point scale for three criteria at each station: construct of interest (e.g., empathy); communication; and overall performance.

### Data collection and analysis

Archival data were collected for each candidate who interviewed between the 2018–2019 and 2021–2022 admissions cycles. Two years of data (2018–2019 and 2019–2020) represented in person MMIs and were aggregated for analysis; two years of data (2020–2021 and 2021–2022) represented vMMIs.

Descriptive statistics for in-person MMIs and vMMIs were calculated for each MMI station. An exploratory factor analysis (principal components analysis) with varimax rotation and Kaiser rule (i.e. retaining factors with eigenvalue > 1.0) was used to explore the construct validity of the 2020–2021 vMMI, as explained above. Only one year of vMMI data was used for the factor analysis since one station was dropped for the 2021–2022 vMMI. Pearson correlation was used to examine correlations between stations and Cronbach alpha was used to determine the internal consistency of each vMMI station. After establishing the construct validity and reliability of the vMMI stations, independent *t*-tests were used to examine differences between in-person MMI and vMMI groups. Cohen’s d was used to determine effect sizes, which reflect the magnitude of the differences between groups and serve as measures of practical significance (e.g., D > 0.8 is a large effect size). Group comparisons and effect sizes were calculated based on the average station score (e.g., average of the three rubric ratings). Continuous data are represented as mean (standard deviation). Statistical significance was established at the α = 0.05 level. All analyses were conducted using Stata, version 17. This study was submitted and determined to be exempt from full review by the University of North Carolina at Chapel Hill Institutional Review Board.

## Results

Of the 1,026 candidates included in the study, 438 (42.69%) completed an in-person MMI and 588 (57.31%) completed a vMMI. Factor analysis indicated that each vMMI station formed a single factor with loads ranging from 0.86 to 0.96 (Table 1). The stations accounted for 91.16% of the total variance. As seen in Table 2, there were weak to negligible intercorrelations between stations (r_p_<0.30) and high internal consistency within each station (α > 0.90, range 0.93 to 0.96).

**Table 1:** Factor Analysis Loadings for vMMI

Factor	1	2	3	4	5	6	7
Station	*Station 7*	*Station 3*	*Station 4*	*Station 5*	*Station 1*	*Station 6*	*Station 2*
	Why UNC	Integrity	Adaptability	Empathy	Teamwork- Giving	Critical Thinking	Teamwork- Receiving
Construct	0.93	0.92	0.92	0.92	0.93	0.91	0.89
Communication	0.90	0.92	0.91	0.89	0.91	0.86	0.88
Overall Performance	0.95	0.96	0.96	0.96	0.94	0.94	0.95
% variance accounted for	28.78	15.17	11.11	9.93	9.34	8.79	8.04

**Table 2:** Intercorrelations and Reliabilities of vMMI Stations

Station	1	2	3	4	5	6	7
Teamwork, giving	(0.94)	0.17	-0.01	-0.03	0.16	0.05	0.14
Teamwork, receiving		(0.93)	0.03	0.14	0.13	0.09	0.20
Integrity			(0.95)	0.14	0.09	0.29	0.12
Adaptability				(0.95)	0.12	0.16	0.18
Empathy					(0.95)	0.24	0.16
Critical Thinking						(0.94)	0.27
Why UNC							(0.96)

vMMI = virtual multiple mini-interview; Cronbach alpha shown in parentheses

As shown in Table 3, the mean and standard deviations for each in-person station were: teamwork-giving, 5.42 (2.30); teamwork-receiving, 5.61 (2.45); integrity, 6.16 (1.50); adaptability, 6.46 (1.33); empathy, 6.41 (1.67); critical thinking, 6.35 (1.50); and why UNC, 6.56 (1.53). Average vMMI scores were: teamwork-giving, 6.26 (1.48); teamwork-receiving, 6.62 (1.43); integrity, 6.60 (1.58); adaptability, 6.62 (1.65); empathy, 6.56 (1.93); critical thinking, 6.52 (1.55); and why UNC, 6.68 (1.65). Differences between in-person and vMMI scores were found for the teamwork-giving (p < .01), teamwork-receiving (p < .01), and integrity (p < .01) stations. Medium effect sizes were found for teamwork-giving (D = 0.44) and teamwork-receiving (D = 0.47) and a small effect size was found for integrity (D = 0.28). No differences were found for other stations and the remaining effect sizes were small.

**Table 3:** MMI and vMMI Station Scores

Station	MMI (n = 438) Mean (SD)	vMMI (n = 588) Mean (SD)	p-value	Cohen’s D
Teamwork, giving	5.42 (2.30)	6.26 (1.48)	< 0.01	0.44
Teamwork, receiving	5.61 (2.45)	6.62 (1.43)*	< 0.01	0.47
Integrity	6.16 (1.50)	6.60 (1.58)	< 0.01	0.28
Adaptability	6.46 (1.33)	6.62 (1.65)	0.10	0.10
Empathy	6.41 (1.67)	6.56 (1.93)	0.18	0.08
Critical Thinking	6.35 (1.50)	6.52 (1.55)	0.09	0.10
Why UNC	6.56 (1.53)	6.68 (1.65)	0.20	0.07

*Independent t-tests used to determine differences between groups, with Cohen’s d to determine effect size;* *Teamwork, receiving station not used in 2021–2022 vMMI; n = 286

## Discussion

Assessing attributes predictive of student success is a complex undertaking for health professions schools [19, 20]. The analyses reported here describe the psychometric properties of a vMMI as an admissions assessment tool and its performance relative to similar in-person MMIs at the UNC Eshelman School of Pharmacy. This is one of the first studies to examine the psychometric properties of a vMMI and the first of its kind in pharmacy education. The findings of this study support the validity and reliability of vMMIs and contribute to a growing body of research exploring this alternative to in-person interviews for health professions education [10–13, 17]. In general, our findings suggest that our vMMI was able to distinguish between the attributes it was designed to assess, providing support for content specificity (i.e., seven distinct factors with high factor loads, majority of variance accounted for, and weak correlations between stations). These results also align with other studies examining the psychometric properties of MMIs [1, 9, 21].

For most attributes, candidate performance was similar regardless of setting, providing support for the use of virtual interviewing as an alternative to in-person interviewing. The findings that several attributes were scored significantly higher in the remote environment warrants further exploration. In virtual environments, the authors believe that candidates may be more relaxed and use more amenable body language, which can influence their performance (i.e., communication and ability to quickly and clearly respond to questions). In addition, some constructs may be more difficult for evaluators to assess in virtual environments. The teamwork stations, for example, demonstrated the largest differences between in-person and virtual performance. However, this difference is not surprising since this station was difficult to reproduce in the virtual environment as candidates were communicating via Zoom, which changed the dynamics and the logistics of this station. Another issue to consider is whether candidates received off camera assistance; however, this was not suspected or detected.

While this study provides support for the use of vMMIs, it does not address other aspects of interviews or interview days that schools often use to both evaluate and recruit prospective students. If schools choose to use vMMIs, additional strategies may be necessary to complement the remote interview, such as offering information sessions to highlight various aspects of the school and program; providing an opportunity for candidates to interact with faculty and leaders of the school independent of the interview; offering a video tour, and providing interaction and an informal question and answer session with current students [14, 15]. Strategically planning opportunities for candidates to connect with these individuals in meaningful ways helps to showcase the culture of the program/school and allows candidates to determine fit [8, 15].

As schools adjust to post pandemic expectations and needs, further consideration should be given to how a vMMI might fit into an institution’s philosophy and strategy for recruitment and admissions. For example, does increasing accessibility, reducing barriers, and improving convenience for candidates and interviewers outweigh the importance of providing an opportunity for the candidate to visit the school in person? Prior to the COVID-19 pandemic, visiting the school in person to assess the fit and culture had been an effective recruitment tool for us, based on candidate feedback. Or, could some combination of in-person and vMMIs be feasible and fair? Schools will need to consider how they might both increase access and provide authentic and informative campus-based experiences [14].

This study suggests that vMMIs can provide valid and reliable information about candidates despite several limitations. First, the single institution sample limits generalizability of results. As more schools implement vMMIs, these results should be considered within this growing body of literature. Second, this study did not examine the variability in vMMI scores associated with interviewer bias and other construct-irrelevant variance, which should be examined in future studies with analyses like the Many-Faceted Rasch Model [6]. In addition, the association between vMMI scores for this cohort and their academic performance in the program remains unclear. Future research will evaluate the relationship between vMMI scores and performance in the curriculum. Ongoing assessment of vMMIs and their use as a tool for identifying qualified applicants will further inform refinements to this approach.

## Conclusion

Evaluating candidates for health professions schools is a complex undertaking. Common strategies for interviews often suffer from interviewer bias, poor instrumentation, and high travel and opportunity costs. The vMMI described in this study demonstrated strong psychometric properties, suggesting that it is a viable alternative to in-person interviewing. Additional research is needed to further explore differences between the two approaches and identify strategies that align with institutional priorities for recruitment and admissions.

## Data Availability

The datasets generated and analyzed during the current study are not publicly available to protect the integrity of the admissions process and applicants but are available from the corresponding author upon reasonable request.
